# Key pre-operative CT parameters for cochlear implantation: a focused radiologic review with surgical relevance

**DOI:** 10.1007/s00234-026-04055-z

**Published:** 2026-06-03

**Authors:** Tsz Ho Chow, Ho Sang Leung, Ka Yue Tam, Horace Cheng, Ann Dorothy King, Wai Tsz Chang, Ka Tak Wong

**Affiliations:** 1 Department of Imaging and Interventional Radiology, Prince of Wales Hospital, Hong Kong, China; 2 Department of Imaging and Interventional Radiology, Chinese University of Hong Kong, Hong Kong, China; 3 Department of Ear, Nose and Throat, Prince of Wales Hospital, Hong Kong, China; 4 Department of Otorhinolaryngology, Head and Neck Surgery, Chinese University of Hong Kong, Hong Kong, China; 5 The Institute of Human Communicative Research, The Chinese University of Hong Kong, Hong Kong, China

**Keywords:** Cochlear implantation, Posterior tympanotomy, Round window, Facial recess, Hearing loss, Computed tomography, Inner ear malformations

## Abstract

**Purpose:**

Cochlear implantation is an established treatment for severe to profound sensorineural hearing loss. Pre-operative computed tomography (CT) of the temporal bone is important for surgical planning, yet variability exists in how key imaging parameters are defined and measured across literature. This review highlights the key CT parameters with direct surgical relevance in cochlear implantation, evaluate current literature to address areas where radiological measurement remains ambiguous or inconsistently reported across studies, and to propose a practical framework to guide radiologists in preoperative CT evaluation for cochlear implantation.

**Methods:**

A narrative review of the literature was performed on PubMed and EmBase in the period of 1 January 2000 to 1 March 2026 focusing on CT-based anatomical parameters relevant to the cochlear implantation with emphasis on its individual steps and relevant surgical impediments.

**Results:**

The review identified key CT parameters relevant to cochlear implantation and proposed a structured neuroradiological framework for pre-operative assessment. This includes a stepwise assessment of the receiver–stimulator bed, mastoid, vascular variants, and posterior tympanotomy corridor, with planning for electrode insertion. Key thresholds identified include facial recess width <3 mm (predicting difficult access), tegmen height <3.5 mm (low-lying), and calvarial thickness <4 mm (risk of inner table breach). Standardised measurement of key parameters, including parasagittal facial recess width, and integration into a structured report were recommended.

**Conclusion:**

Preoperative temporal bone CT provides essential anatomical information for cochlear implantation. Standardisation of parameter definitions and measurement techniques may enhance radiologic reporting, facilitate surgical planning, and reduce operative risk. A structured, surgically oriented CT assessment framework improves interdisciplinary communication.

## Introduction

Hearing impairment is a common and significant disability worldwide [1]. Cochlear implantation is indicated in patients with severe to profound sensorineural hearing loss who receive limited benefit from conventional hearing aids. Traditionally hearing loss is defined as severe and profound when pure-tone thresholds exceed 71–95 dB and 95 dB, respectively. Cochlear implantation is also considered in selected patients with residual low-frequency hearing [2]. The procedure involves the surgical placement of an electrode through the mastoid and middle ear into the cochlea, which carries risks particularly to the facial nerve [3, 4].

The use of pre-operative imaging, particularly computed tomography, is essential for surgical planning to assist patient selection and avoid surgical complications [5]. While previous reviews on this topic have described a variety of pathologies on imaging [6–8], there has been less emphasis in the imaging parameters that directly impact the surgical approach and difficulties in electrode placement. Based on previous studies, this article will summarise the CT findings with an emphasis on imaging parameters along the electrode placement pathway, from the scalp to the cochlear duct, that are important in preoperative CT evaluation (Table 1). Based on these imaging parameters, we propose a structured neuroradiological reporting template for pre-operative CT assessment in cochlear implantation (Appendix) for systematic assessment of the receiver–stimulator bed, mastoid, vascular variants, posterior tympanotomy corridor, planning for electrode insertion and inner ear malformation.

**Table 1:** Summary of key pre-operative CT imaging parameters for cochlear implantation, with their suggested radiological assessment methods based on previous studies, surgical relevance and evidence basis

Imaging Parameter	Radiological Assessment	Surgical Relevance	Evidence Basis
Scalp thickness	Measured at implant site (~ 30–45° and 5 cm posterosuperior to EAC)	Thick scalp may impair transmission of electrical signal and magnetic coupling; thin scalp may lead to skin necrosis	Prospective cohort study [9], retrospective cross-sectional study [10]
Calvarial thickness	Axial measurement at implant site. Thin calvarium <4 mm.	Thin calvarium risks dural or venous sinus injury safe drilling and secure fixation.	Retrospective cohort studies [11, 12]
Mastoid pneumatisation	Degree or extent of aeration graded in Likert scale (well, moderate and poor aeration)	Small and sclerotic mastoid limits surgical space and access to facial recess, risks injury to adjacent structures.	Prospective observational study [13], retrospective cohort study [14]
Vascular anomalies	Presence of enlarged mastoid emissary vein (> 3 mm), high riding jugular bulb (above level of cochlear basal turn, IAC or EAC)	Risk of vascular injury	Retrospective cohort studies [14–16]
Tegmen tympani height	Vertical distance between tegmen and lateral semicircular canal axis. Low-lying tegmen < 3.5 mm.	Low-lying tegmen increases surgical difficulty and risk of dural injury.	Prospective observational study [13]
Facial recess width	Perpendicular distance between mastoid FN canal and chorda tympani on parasagittal plane at the widest portion of facial recess, or between FN and posterior EAC wall at level of RW and cochlear basal turn on axial plane. Narrow recess <3 mm.	Narrower recess restricts surgical access during posterior tympanotomy and RW exposure, with higher risk of facial nerve or chorda tympani injury.	Prospective cohort study [17], retrospective cohort study [14]
Round window	Size, morphology and orientation (including hypoplasia and bony overhang)	Unfavourable anatomy reduces RW exposure which may require drilling or even cochleostomy.	Retrospective cohort study [18], cadaveric anatomical study [19]
Cochlear duct length	CDL is estimated using software reconstruction or manually using the mathematical formula: *CDL = 4.16 × A – 4* to convert the cochlear diameter (A-value which is measured from the centre of round window through the modiolus axis, to the lateral wall of basal turn) to the length	Determines electrode selection and insertion depth	Retrospective cohort study [20], cross-sectional expert survey (global consensus) [5]
Incomplete partition anomalies	Cochlear internal architecture (modiolus and interscalar septa) and external contour	Increased risk of CSF gusher, perilymphatic fistula and meningitis.	Systematic review of retrospective studies [21, 22]
Cochlear hypoplasia	Cochlear morphology and size. Cochlear hypoplasia is considered if basal turn length < 7.5 mm and mid-modiolar height < 3.4 mm.	Smaller cochlea requires shorter electrode arrays or less insertion depth.	Retrospective cohort study [23]
Vestibular aqueduct	Enlarged VA is defined as midpoint diameter > 1.0 mm or opercular diameter > 2.0 mm (Cincinnati criteria), or midpoint diameter > 1.5 mm (Valvassori criteria).	Enlarged VA is associated with an increased risk of intraoperative cerebrospinal fluid gusher and postoperative meningitis, and may influence electrode choice.	Systematic review of retrospective and prospective studies [24]

*EAC* external acoustic canal, *IAC* internal acoustic canal, *FN* facial nerve, *RW* round window, *CDL* cochlear duct length, *CSF* cerebrospinal fluid, *VA* vestibular aqueduct

## Imaging protocol

### Modality

According to the 2026 global consensus on preoperative imaging for cochlear implantation, CT and MRI are complementary modalities: CT for surgical planning, and MRI for diagnosis and assessing operability [5].

Multi-slice computed tomography is essential for evaluation temporal bone anatomy prior to cochlear implantation. Cone-beam CT, if available, is an alternative CT technique that offers excellent spatial resolution for osseous structures and a lower radiation dose, which is particularly beneficial to paediatric patients. However, CBCT is more susceptible to motion artefact and has limited soft tissue contrast [5].

Magnetic resonance imaging (MRI) complements CT by allowing assessment of the vestibulocochlear nerve and its branches, and brainstem. It does not involve ionising radiation, which again is advantageous in children. However, MRI provides less details of osseous structures and may require sedation or general anaesthesia in paediatric patients [5]. Emerging MRI techniques such as “black bone” MRI for improved visualisation of osseous structures have been reported [9].

### Scanning protocol

**Fig. 1:**
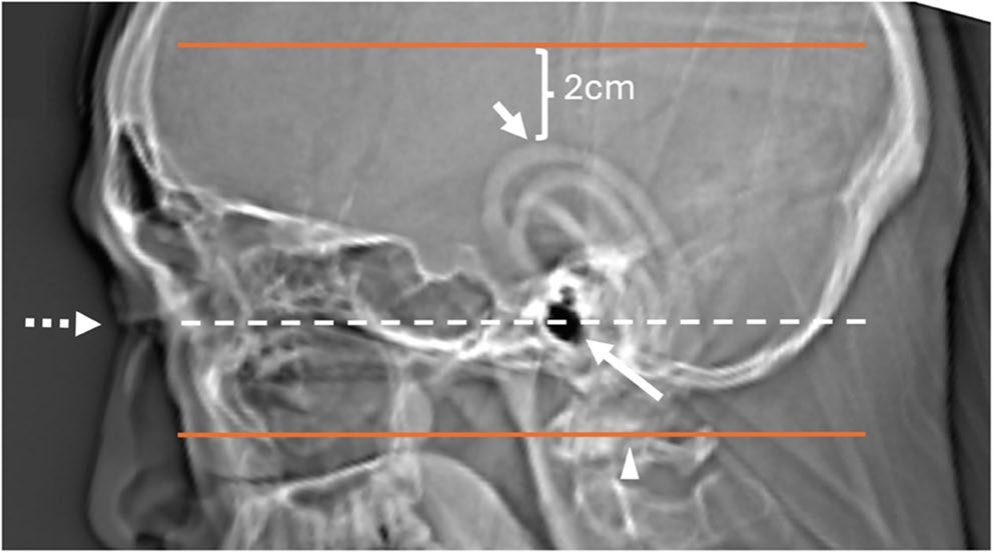
Reference axial plane and scan range. Scout image showing the canthomeatal (orbitomeatal) line (dashed line) between the lateral canthus (dashed arrow) and external auditory canal (long solid arrow), which serves as the reference plane for axial acquisition. Scan coverage (solid lines) may be extended from approximately 2 cm above the superior tip of auricle (short solid arrow) to the inferior mastoid tip (arrowhead), ensuring inclusion of all key temporal bone landmarks for cochlear implant planning

The axial plane of temporal bone CT is typically defined parallel to the orbitomeatal line (Fig. 1), although several variations have been described in the literature. The canthomeatal line is drawn between the lateral canthus and the centre of the external auditory meatus. Its advantage is that it can be easily identified clinically and allows bedside adjustment of gantry tilt to standardise the axial plane across patients [10, 11]. Alternatively, some authors adopt the supra-orbitomeatal or infra-orbitomeatal lines, drawn between the external auditory meatus and the orbital roof or orbital floor, respectively [12–14]. These landmarks are less practical to identify at the bedside but can be recognised on CT scout images, thereby providing more reproducible skull base orientation. An additional advantage of scanning parallel to the supra-orbitomeatal plane is the avoidance of direct radiation exposure to the lens, which is particularly relevant in paediatric patients.

**Fig. 2:**
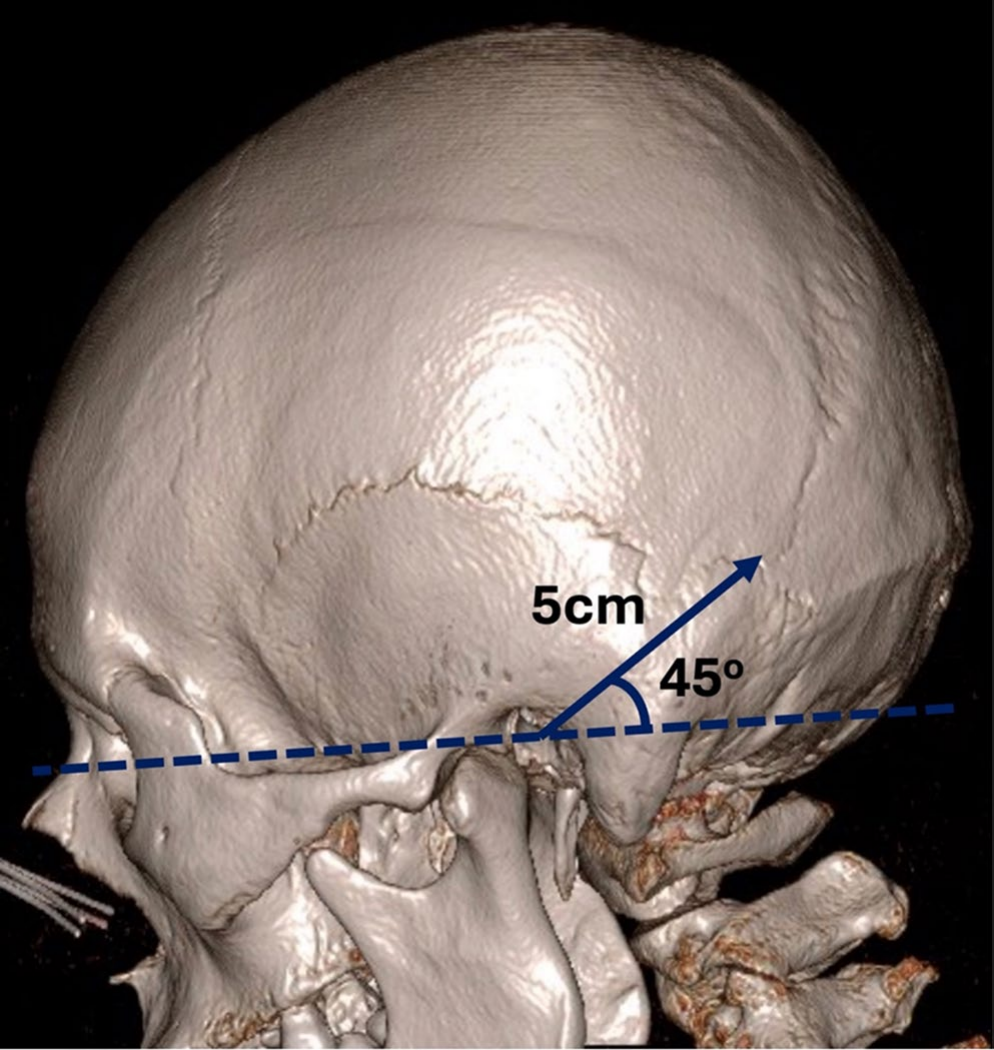
Expected site of receiver–stimulator placement. 3D skull reconstruction showing the typical site of receiver well drilling, located approximately 5 cm posterosuperior to the external auditory canal and angled 30–45°

The CT scan range should include the entire petrous temporal bone and the anticipated site of cochlear implantation, including the region of bony well drilling, typically located 30–45° and 5 cm posterosuperior to the external auditory canal (EAC) (Fig. 2). The scan range for preoperative temporal bone CT is not explicitly defined in the existing review articles [6–8]. Although review article by Connor suggested that the scan volume should cover approximately 5 cm posterosuperior to the EAC [15], the measurement may vary depending on the orientation of the axial scanning plane. The site of bony well drilling is often situated higher than the pinna and may not be included from standard scan protocols.

The inferior scan extent should include the mastoid tip. For the superior extent, extending the scan range to approximately 2 cm above the superior tip of the auricle (Fig. 1) may improve inclusion of the anticipated surgical site; however, this is associated with an increase in radiation dose. A limitation is that quantitative dosimetry data specific to this adjustment are lacking in the literature, as the magnitude of dose increase will depend on scanner type and acquisition parameters. Therefore, the decision to extend the superior scan extent should balance the potential surgical benefit against the radiation risk.

Following image acquisition, these are usually reconstructed in thin slices (0.6 mm or less) under bone algorithm to enable multiplanar reconstruction and detailed evaluation of surgical access.

## Surgical procedure of cochlear implantation and relevant anatomical parameters on CT

Cochlear implantation surgery involves several key surgical components; each has its own caveats relevant to the surgical procedure and anatomical structures involved. In this section we will detail the surgical relevance of pre-operative imaging in a stepwise manner.

### Surgical incision and creation of receiver well

Cochlear implantation begins with a postauricular incision (around 4 cm or longer) to expose the mastoid cortex. The internal receiver–stimulator is positioned posterosuperior to the incision, typically within a subperiosteal pocket created in the outer calvarial cortex or a shallow bony well [16].

### Imaging parameters and surgical relevance

#### Scalp thickness

**Fig. 3:**
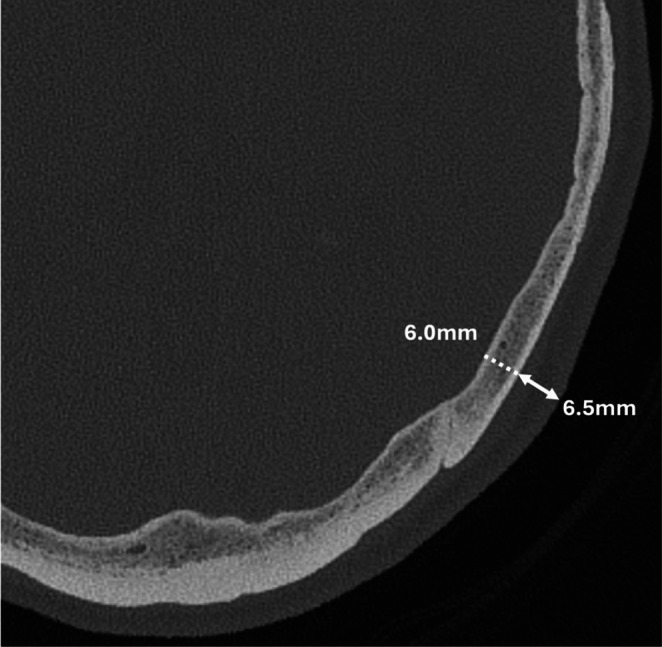
Scalp and calvarial thickness. Axial CT demonstrating measurement of scalp thickness (double arrow) and calvarial thickness (dashed line). These parameters are important for incision planning, secure fixation of the receiver well, and avoid dural injury

Scalp thickness is considered in surgical planning of the skin flap overlying the receiver-stimulator (Fig. 3). A thick scalp may reduce the transmission of electrical signals between external and internal components, and theoretically the magnetic coupling between the external and internal components of the device. Some centres may perform intraoperative thinning of the soft tissue flap (e.g., defatting or muscle debulking) in selected cases, but this is less of a problem with the availability of stronger magnets in modern cochlear implantation [17, 18]. On the other hand, rarely a very thin scalp has been associated with the risk of skin necrosis due to mechanical pressure by the magnets.

In the past, scalp thickness was measured intraoperatively by inserting a sterile needle perpendicular to the skull [17]. Currently, CT or MRI can be used to evaluate scalp thickness to provide preoperative information that will aid planning of the surgical approach [19]. Measurements are typically performed at the anticipated implant site — approximately 30–45° and 5 cm posterosuperior to the external auditory canal [15]. While optimal scalp thickness varies across manufacturers, a commonly accepted upper limit is 6–7 mm, above which surgical thinning of the skin flap may be required [17, 18].

#### Calvarial thickness

The subperiosteal pocket technique, without drilling a bony well, is commonly used for fixation of the receiver-stimulator due to its thin profile. However, this fixation method varies among individual centres, and in some cases, a bony well may be drilled in conjunction with the subperiosteal pocket to enhance device stabilisation [16]. In such cases, calvarial thickness will be important for the safety and feasibility of drilling (Fig. 3). A thin calvarium may increase the risk of dura or venous sinus injury during drilling, whereas adequate bone thickness permits secure fixation without breaching the inner table. In addition, the placement of the receiver–stimulator should be selected based on the flatness of the temporal bone to ensure stable seating of the device [20].

Calvarial bone thickness can be measured on axial CT at the anticipated implantation site. This is especially relevant in paediatric patients, where the skull is usually thinner than in adults, with average thickness ranging from approximately 2.9 mm in children under 5 years to 6.7 mm in older children [21]. Modern cochlear implant receivers can be as thin as 3.9 mm [22, 23]. A calvarial thickness of at least 4 mm is preferred to allow safe drilling and secure fixation while minimising the risk of inner table breach or dural injury [24].

#### Mastoid emissary vein

**Fig. 4:**
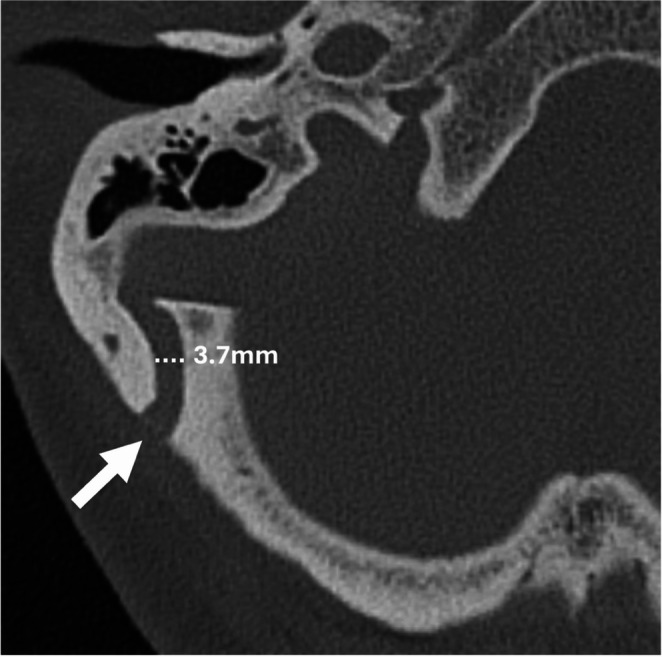
Enlarged mastoid emissary vein. Axial CT demonstrating an enlarged mastoid emissary vein (arrow) connecting the sigmoid sinus with extracranial veins, which should be recognised to avoid inadvertent injury

Mastoid emissary vein (MEV) represents accessory venous channels connecting the sigmoid sinus with extracranial veins and are usually directed posteriorly towards the occiput (Fig. 4). The definition of enlarged MEV varies in the literature with proposed cut-off thresholds of between 2.0 and 3.5 mm [25, 26]. Enlarged MEVs may occasionally be encountered near the receiver–stimulator site, although intraoperative bleeding from these veins can be managed by cauterisation, a clear pre-surgical guide could be helpful to avoid inadvertent venous transgression.

Another related but rare venous anomaly is the persistent petrosquamosal sinus, an embryonic venous channel that connects the transverse or sigmoid sinus to the extracranial venous system through the temporal bone and may persist in congenital conditions, particularly CHARGE syndrome [27]. Recognition of this variant on preoperative imaging is important to prevent significant venous bleeding.

### Cortical mastoidectomy

After incision, mastoidectomy is performed to create a surgical corridor to the middle ear [28]. In cochlear implantation, a limited cortical mastoidectomy is usually sufficient, without need of full exenteration all mastoid air cells as in middle ear disease surgery. The supra-meatal (MacEwen) triangle, a small bony depression on the lateral temporal bone, serves as a superficial landmark for entry [29]. Care should be taken in the process to avoid injury to critical structures, including the mastoid segment of the facial nerve, sigmoid sinus, and vascular variants such as an enlarged mastoid emissary vein.

### Imaging parameters and surgical relevance

#### Mastoid pneumatisation and pathology

**Fig. 5:**
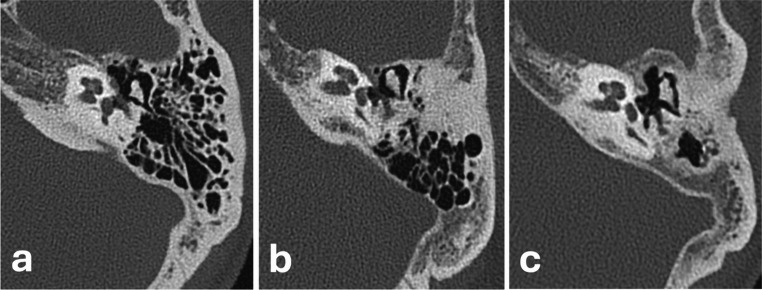
Mastoid pneumatisation. Axial CT showing well-pneumatised (**a**), moderately pneumatised (**b**) and poorly pneumatised (**c**) mastoid air cells, the latter of which may increase surgical difficulty during cortical mastoidectomy by limiting working space, hence increasing risk of facial nerve injury

Mastoid pneumatisation varies among individuals, and both qualitative and quantitative classification systems have been described. Qualitatively, the three-tier system based on visual assessment of mastoid air cell density is most commonly adopted: (1) poorly pneumatised (sclerotic) mastoids with minimal or absent air cells, (2) moderately pneumatised (diploic) mastoids with a mixed bone–air appearance, and (3) well-pneumatised (pneumatic) mastoids with extensive, confluent air cells (Fig. 5) [30, 31]. Pneumatisation has also been graded according to its anatomical extension relative to the sigmoid sinus [32]. Quantitative approaches include CT volumetry of mastoid air cell volume and percentage estimation of pneumatisation within the mastoid temporal bone [33]. While quantitative methods may provide more precise measurements, qualitative classifications are simpler, widely used in previous cochlear implant studies [34, 35], and seem sufficient for assessing surgical difficulty in clinical setting.

Reduced mastoid pneumatisation and more importantly smaller mastoid size may be more surgically challenging. Small mastoids reduce surgical working space and restrict access to the facial recess, while poorly pneumatised mastoid requires additional bone drilling. These increase the risk of injury to adjacent structures such as facial nerve, sigmoid sinus, and dura, thereby making cortical mastoidectomy more challenging [34, 35].

Pathology within the mastoid air cells is also important to recognise, including mastoid effusion, cholesteatoma, or granulation tissue from prior inflammation. Such abnormalities can alter the operative field and increase the risk of postoperative infection or device-related complications, leading to explantation [36]. In patients with chronic otitis media or cholesteatoma, eradication of disease is generally recommended prior to cochlear implantation. MRI with diffusion-weighted imaging is often helpful in differentiating cholesteatoma from effusion and guides management strategies, which may include tympanomastoid surgery and antibiotics for clearance of infection, either followed by delayed cochlear implantation or performed as part of a staged procedure [37].

#### Vascular anomalies

**Fig. 6:**
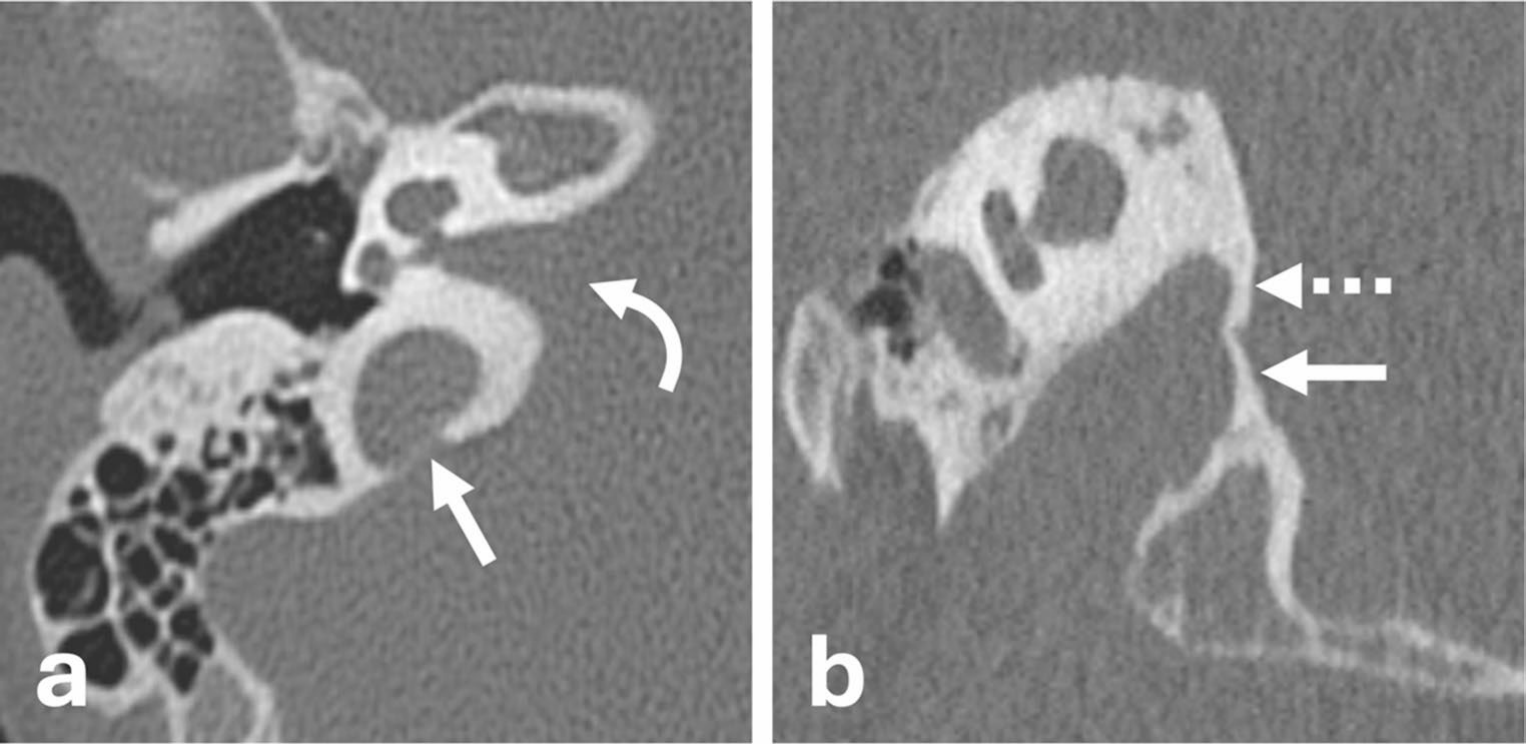
High-riding jugular bulb with diverticulum. (**a**) Axial CT showing a high riding jugular bulb (straight arrow) at the level of internal acoustic canal (curved arrow). (**b**) Sagittal CT showing a diverticulum (dashed arrow) at the tip of the high riding jugular bulb (solid arrow)

Vascular anomalies of the temporal bone include high-riding jugular bulb (i.e. superior margin reaching level of the floor of internal acoustic canal) or dehiscent jugular bulb with or without diverticulum (Fig. 6). They are important to identify preoperatively due to increased risk of intraoperative haemorrhage [38], particularly when they lie close to the surgical field tracking from the MacEwen’s triangle laterally to facial recess medially. High-riding jugular bulb (HJB) is variably defined as the jugular bulb extending above the level of the cochlear basal turn, internal or external auditory canal, while jugular bulb dehiscence (JBD) refers to deficiency of the sigmoid plate separating the bulb from the middle ear cavity [39]. Both anomalies are surgically relevant as they increase the risk of vascular injury and bleeding [39], and in rare cases, HJB may overlie the round window hence obstructing electrode insertion [38].

#### Height of tegmen tympani

**Fig. 7:**
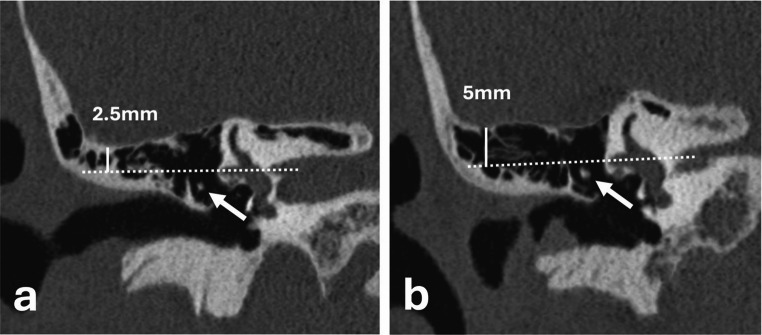
Height of the tegmen tympani. Coronal CT showing measurement of tegmen height (solid lines) above the axis of the lateral semicircular canal (dashed lines), at the level of incus short process (solid arrows). (**a**) Low-lying tegmen is associated with increased surgical difficulty and risk of dural injury. (**b**) CT of another patient with normal tegmen height for comparison

Height of tegmen tympani is an important imaging parameter that can help predict the technical difficulty of cortical mastoidectomy. The tegmen tympani is a thin bony plate forming the roof of the middle ear cavity, separating it from the middle cranial fossa. Tegmen height is typically measured on coronal CT images as the perpendicular distance between the lowest point of the tegmen tympani and the lateral semicircular canal on the coronal plane (Fig. 7) [6, 30, 40]. A tegmen height of less than 3.5 mm is considered low-lying and is associated with higher surgical difficulty. The reduced height makes it more challenging to identify key anatomical landmarks, such as lateral semicircular canal and incus body, which are essential for localising the facial nerve [30]. It is also associated with a higher risk of dural exposure and potential complications such as cerebrospinal fluid leak or pneumocephalus [40].

#### Surgical approaches alternative to mastoidectomy

Suprameatal approach may be considered in patients with unfavourable access through conventional posterior tympanotomy, including poor mastoid pneumatization or aberrant facial nerve. With less need for surgical exposure of the mastoid segment of facial nerve and chorda tympani and reducing chance of injury, the trade-off of supramastoid approach include restricted visualisation of the round window and potential difficulty in electrode positioning [41]. Assessment of tegmen height is essential, as a low-lying tegmen may pose risk of dural breech and CSF leak [42].

Transcanal approach is another option which accesses the round window via the external auditory canal (EAC). One technique involves raising a tympanomeatal flap with creation of a bony groove along the EAC to accommodate the electrode array. This approach requires minimal mastoid drilling but is less commonly adopted due to risks of tympanic membrane perforation, electrode exposure, and extrusion. An alternative transcanal technique involves removal of the entire EAC skin and tympanic membrane, followed by blind sac closure of the EAC opening. While this provides a direct surgical corridor and reduces the risk of electrode exposure, it carries a risk of EAC cholesteatoma formation [43]. Pre-operative CT should evaluate along the surgical corridor including EAC, middle ear, and round window orientation [44]. Other less commonly adopted alternatives, including middle cranial fossa approach or robotic assisted surgery to middle ear access, have also been reported [45].

### Posterior tympanotomy

Posterior tympanotomy is performed after cortical mastoidectomy. The first step of posterior tympanotomy is to identify the bony covering of lateral semicircular canal which is followed by careful bone drilling to visualize the incus and fossa incudis, which form the superior border of facial recess [46]. Subsequently, meticulous bone drilling by diamond burr and under constant irrigation allows identification of mastoid segment of facial nerve and chorda tympani [28], which form the posterior and anterior walls of the facial recess respectively. Entrance into the middle ear cavity is then performed through this facial recess to allow visualisation of the round window niche for electrode insertion.

### Imaging parameters and surgical relevance

#### Facial recess width

CT provides valuable assessment of the facial recess, as a narrow facial recess helps predict the difficulty of posterior tympanotomy and round window visibility [3, 4, 47–49].

**Fig. 8:**
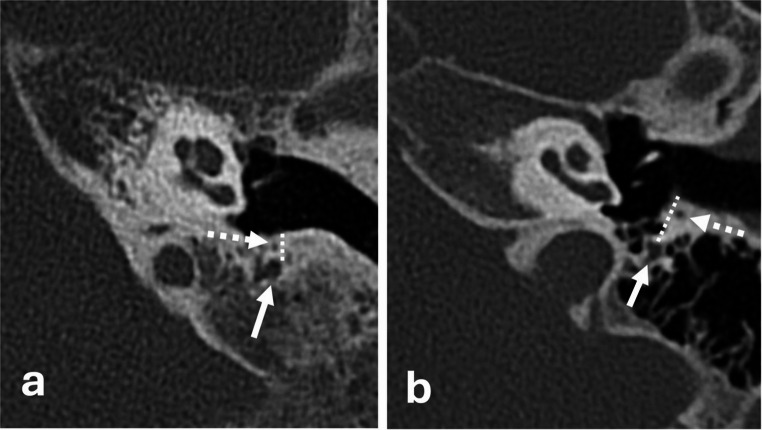
Axial measurement of facial recess width (FRW). Axial CT showing FRW (dashed lines) between the facial nerve canal (solid arrows) and the posterior wall of external auditory canal at the level of round window (RW) and cochlear basal turn. A narrow facial recess (**a**) (1.5 mm) is associated with increased difficulty of posterior tympanotomy and reduced RW visibility, when compared to a normal FRW (**b**) (4 mm). Chorda tympani (dashed arrows) are also identified

The facial recess width (FRW) is the more commonly assessed parameter on CT although significant variability exists in its measurements. On axial CT, FRW may be measured from the mastoid portion of facial nerve canal to either the chorda tympani canaliculus [47, 48], the tympanic annulus or posterior wall of external acoustic meatus (EAC) (Fig. 8) [10, 12, 13, 50, 51].

There is currently no standardised axial plane or level at which FRW should be measured. As the facial recess has an inherent inverted triangular configuration with the facial nerve and chorda tympani diverge cranially, a difference of up to 1.9 mm in measurements have been reported [52], it is therefore important to standardize the plane of measurement.

Some radiological studies measured FRW at the level of the round window [10, 12], while others do not define a specific reference level. This inconsistency contributes to wide variability in reported FRW values [10, 12, 13, 47, 48, 50, 51], making it difficult to establish a reliable cut-off for predicting surgical access. Nonetheless, the commonly quoted FRW <3 mm would suggest difficult access and higher risk of facial nerve injury [34, 53].

**Fig. 9:**
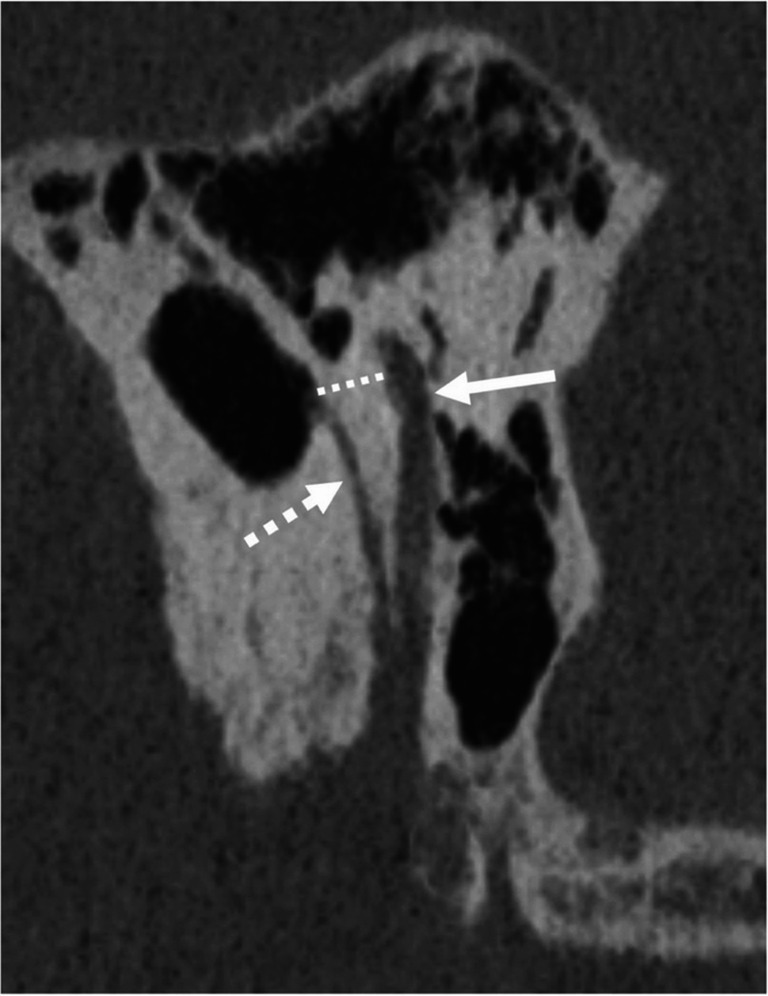
Parasagittal measurement of facial recess width (FRW). Oblique-sagittal reformatted CT demonstrating FRW (dashed lines) as the perpendicular distance between the mastoid facial nerve canal (solid arrows) and the chorda tympani (dashed arrows) at the level of the widest portion of the facial recess

Alternatively, we recommend measurement of FRW on parasagittal reformatted CT as the perpendicular distance between the facial nerve canal and the chorda tympani at the level of the widest portion of the facial recess, using an oblique-sagittal plane that depicts both structures on the same slice (Fig. 9). This approach is surgically relevant as it reflects the anatomical corridor used during posterior tympanotomy and provides a reproducible reference for assessment. However, the main limitation of this measurement method is that published data on inter- and intraobserver reliability are lacking, and further validation studies are required to establish its reproducibility. In addition, direct visualisation of the chorda tympani on conventional CT can be challenging because of its very small calibre (posterior canaliculus < 0.5 mm) and anatomical variation [54]. In such cases, indirect anatomical landmarks related to the course of the chorda tympani, such as the tympanic annulus or the posterior wall of the external auditory canal, may be used for measurement of FRW on axial CT [6]. Alterative CT techniques, such as cone-beam CT (CBCT) and ultra-high-resolution CT (ultra-HRCT) may also be helpful. CBCT provides better delineation of fine osseous structures [5], while ultra-HRCT has also been described for FRW measurement, although its use remains limited by cost and local availability [55].

### Electrode insertion

Both the site and length of cochlear implant electrode insertion are important for effective electric stimulation of the cochlear neural elements. Round window and cochleostomy approaches are the two most common adopted methods for electrode insertion. Round window approach is often preferred if surgically feasible, which allows closer positioning of the electrode to the modiolus for effective stimulation of cochlear neural elements and less bone drilling and trauma to hearing structures for better hearing preservation [56]. Cochleostomy involves drilling a separate opening in the cochlear promontory anteroinferior to the round window which is adopted when the round window is obscured or inaccessible.

Subsequently, a predetermined length of cochlear electrode is inserted into the scala tympani, with the tip aiming at 1–1.5 turns within the cochlea (i.e. 360–450-degree insertions). Estimation of cochlear duct length (CDL) is important to guide electrode selection and insertion depth.

After electrode insertion, intraoperative telemetry is used to assess impedance and confirm device integrity. After ensuring that no potential pathway for meningitis remains, the procedure is completed with closure of the periosteal and skin layers.

### Imaging parameters and surgical relevance

#### Round window

CT allows assessment of round window (RW) parameters, which is crucial for predicting insertion difficulty and selecting the appropriate surgical approach. Anatomical variations in RW size, morphology, and angulation can significantly impact surgical visibility and accessibility.

**Fig. 10:**
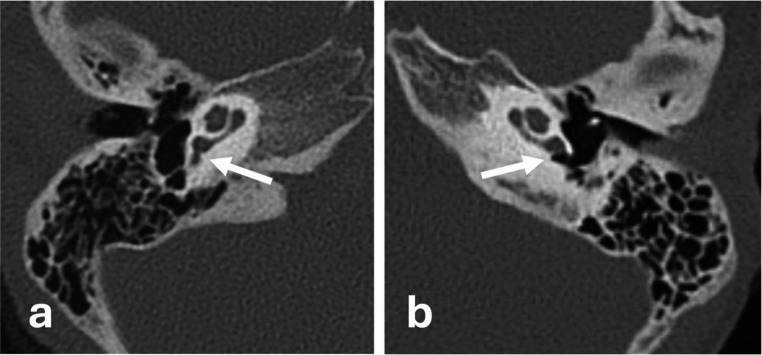
Round window hypoplasia. (**a**) Axial CT showing hypoplastic round window which may limit access for electrode insertion and necessitates cochleostomy approach, as opposed to (**b**) a normally developed round window

- **Size**: RW diameter < 1.5 mm is considered narrow and may restrict electrode insertion (Fig. 10) [57], particularly when the exposed membrane is smaller than the electrode’s apical diameter. In such cases, a promontory cochleostomy may be a more suitable alternative to round window insertion [58].
- **Morphology**: The shape of the round window niche influences its visualisation. An incomplete bony overhang at the round window niche (i.e. C-shaped niche) is more frequently associated with adequate exposure than those with complete overhang (O-shaped niches), the latter may require additional drilling to improve surgical visibility [59, 60].
- **Angle of Cochlear Basal Turn**: Defined as the angle between the axis of the cochlear basal turn and the cranial sagittal midline, with a steep angle (> 58.5°) reported to be associated with reduced or partial RW exposure on posterior tympanotomy [61].

In addition, the CT facial recess view has been proposed as a practical method to assess RW accessibility. This view evaluates the position of the RW relative to the mastoid segment or second genu of the facial nerve. Posteriorly located round windows are associated with difficult surgical access [62].

#### Cochlear duct length

**Fig. 11:**
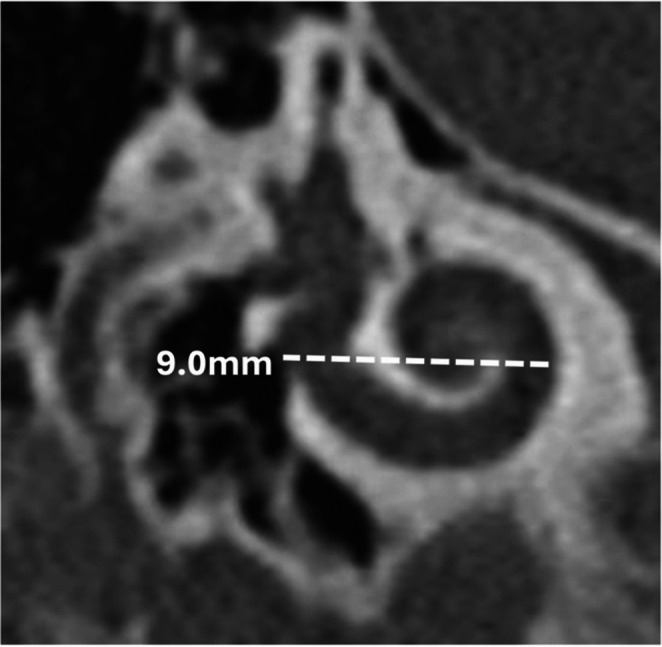
Cochlear diameter. Oblique sagittal CT demonstrating measurement of the cochlear diameter (dashed line), defined as the distance from the centre of the round window to the opposite outer wall of the basal turn. Cochlear duct length can be estimated based on cochlear diameter, which is important for guiding electrode selection and insertion length

Cochlear duct length (CDL), defined as the length of the scala media, is important for cochlear implant planning. Deeper insertions can improve speech perception by stimulating the apical cochlea [63], whereas excessive depth increases the risk of intracochlear trauma [64]. Although no universally accepted measurement standard exists, two main imaging-based approaches are described:

1. **Mathematical formula estimation**: Alexiades et al. proposed the formula *CDL = 4.16 × A – 4* to estimate the cochlear duct length at the level of the organ of Corti, where the A-value represents the greatest distance from the centre of the round window through the modiolar axis to the lateral wall of the basal turn, i.e. cochlear diameter (Fig. 11) [65, 66]. This method is simple and requires only a single linear CT measurement.
2. **Software-based approaches**: These include CT-based 3D multiplanar reconstruction software, which allow direct tracing of scala tympani to determine CDL [67], as well as otosurgical planning software such as Otoplan, which uses semi-automated measurements of cochlear dimensions and applies geometric models to calculate CDL that are comparable to manual measurements [68, 69]. In clinical practice, if available, software-based methods are preferred [5], as they provide higher accuracy and inter-rater reliability when compared to mathematical estimation [67]. The limitations include being more time-consuming and requiring specialised expertise or dedicated software.

#### Inner ear malformations

Congenital inner ear malformations (IEM) may influence surgical candidacy, electrode selection, and risk of complications [70, 71]. Cochlear aplasia represents a complete absence of the cochlea, while rudimentary otocyst is defined as a small otic capsule remnant. In both conditions, cochlear implantation is contraindicated, and auditory brainstem implantation is the treatment of choice.

**Fig. 12:**
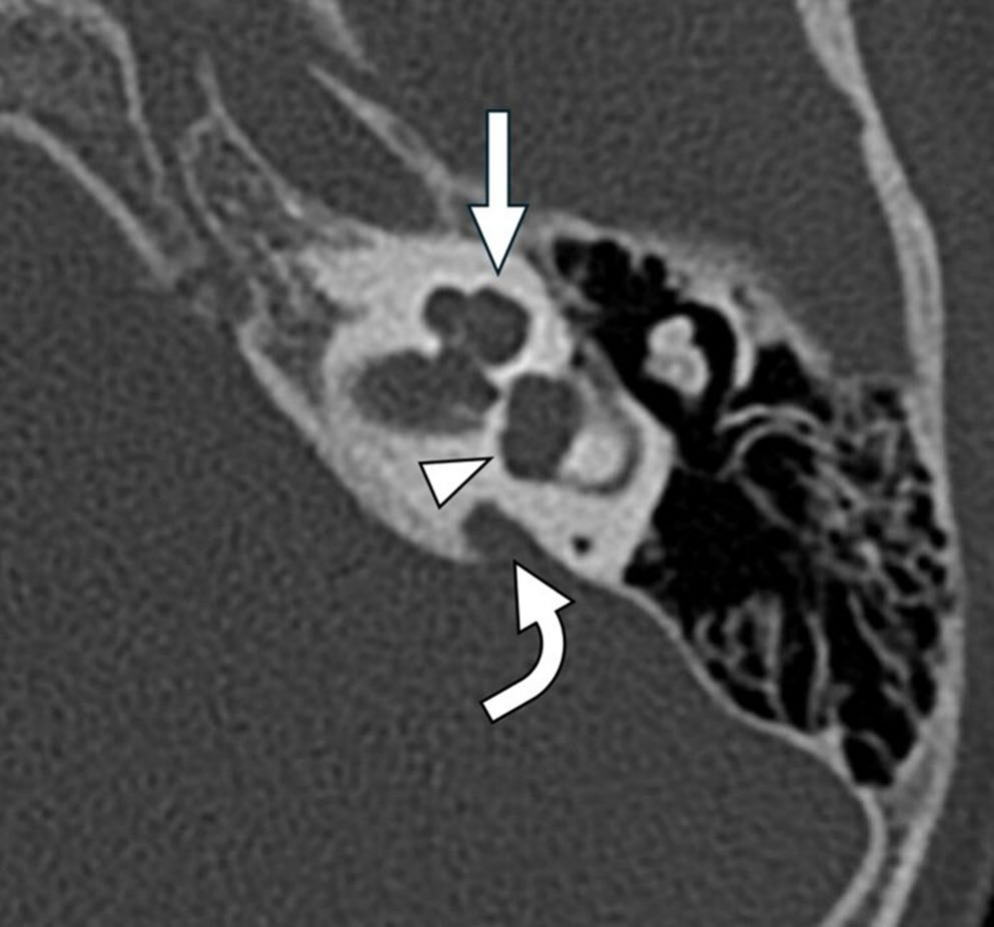
Incomplete partition type II. Axial CT showing a cystic cochlear apex (arrow), enlarged vestibule (arrowhead), and dilated vestibular aqueduct (curved arrow), consistent with incomplete partition type II

**Fig. 13:**
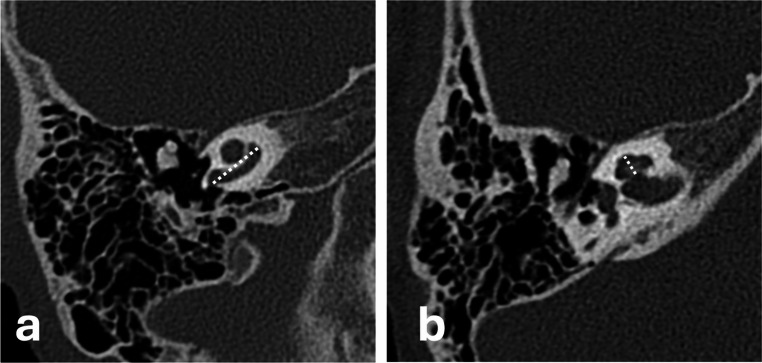
Measurement parameters of cochlea on axial CT images for cochlear hypoplasia. (**a**) Basal turn length is measured along the basal turn of cochlea at the level of round window. (**b**) Mid-modiolar height is measured as the distance from cochlear apex to the base of modiolus. Cochlear hypoplasia is considered when the basal turn length and mid-modiolar height measure less than 7.5 mm and 3.4 mm respectively

**Fig. 14:**
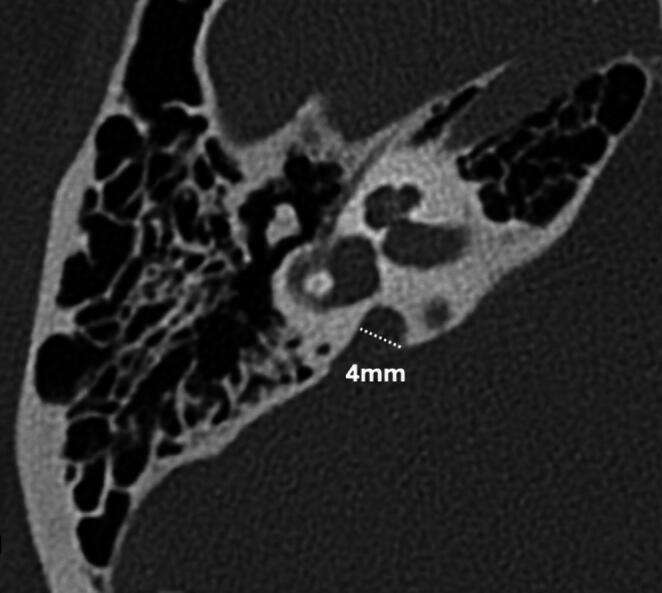
Enlarged vestibular aqueduct (EVA). Axial CT showing an enlarged vestibular aqueduct with opercular diameter > 1.9 mm. EVA is associated with an increased risk of cerebrospinal fluid gusher during cochlear implantation

In the milder spectrum of IEMs, surgical modifications are often required together with the presence of particular surgical hazards as follow:

A **Common Cavity Malformation**
  Common cavity malformation is characterised by failure of differentiation between the cochlea and vestibule, resulting in a single ovoid or round cystic cavity without internal partitioning. The internal auditory canal (IAC) typically enters centrally into the cavity [70], usually smaller and maldeveloped necessitating individualized electrode selection estimated based on its circumference on pre-operative CT [72]. The condition may be also associated with an absent cochlear nerve contributing to a contraindication for cochlear implantation, which should be scrutinized on MRI. Upon surgery, specific complications including cerebrospinal fluid (CSF) gusher, perilymphatic fistula, and meningitis may also occur, due to abnormal communication between the cavity and the IAC [73, 74].
B **Incomplete Partition (IP) Anomalies**
  IP anomalies type I–III involve abnormal internal cochlear architecture with preserved external contours. IP-I shows a cystic cochlea without modiolus or interscalar septa. IP-II has 1.5 cochlear turns with fused middle and apical turns and defective apical modiolus, often with enlarged vestibular aqueduct (Fig. 12). IP-III lacks the modiolus but retains interscalar septa and is associated with X-linked deafness [70]. Quantitative measurements for diagnosis of IP-II on CT is lacking; a ≥ 1.2 mm distance between the osseous spiral lamina and basilar membrane on CT (i.e. distance X) has been proposed on MRI [75] although these structures are difficult to be visualized on CT. The presence of abnormal communications through defective modiolus and cochlear aperture would result in risk for CSF gusher, perilymph fistula or meningitis [76], although perilymphatic fistula and recurrent meningitis are more common in IP-I [73, 77] while CSF gusher more common in IP-III [74].
  On pre-operative CT, qualitative assessment of cochlear basal turn morphology has been proposed. A width-to-length ratio ≥ 0.75 suggests a more rounded configuration, whereas lower ratios indicate an elliptical morphology [78]. Variations in basal turn shape (e.g. circular or elliptical forms) may influence electrode trajectory and intracochlear positioning [79].
C **Cochlear Hypoplasia**
  Cochlear hypoplasia (CH) refers to a small cochlea with varying degrees of modiolar and scalar development and is classified into types I–IV based on morphology. CH-I appears as a small bud-like cochlea with absent modiolus and interscalar septa. CH-II demonstrates a cystic hypoplastic cochlea with a normal external outline but deficient internal architecture, predisposing to cerebrospinal fluid gusher and electrode misplacement. CH-III is characterised by a cochlea with fewer than two turns, maintaining a similar external contour to a normal cochlea but with fewer turns, short modiolus and shortened interscalar septa. CH-IV shows hypoplastic middle and apical turns but normal basal turn [70]. Measurement parameters of the cochlea, including length of basal turn measured on axial CT images at the level of round window (basal turn length), and the perpendicular distance from the cochlear apex to the base of modiolus (mid-modiolar height), are helpful for diagnosis of types I to III cochlear hypoplasia, with a cut-off of 7.5 mm and 3.4 mm for basal turn length and mid-modiolar height respectively. (Fig. 13) [80]. Differentiation of the more subtle cochlear hypoplasia type IV from normal could be challenging: a shorter cochlear duct length along its lateral relative to normal patients have been observed but a specific cut-off is not validated [80]. MRI is also required to assess the presence of the cochlear nerve. Detection of cochlear hypoplasia is important as it may require a shorter electrode to adopt for reduced insertion depth [79].
D **Vestibular Aqueduct**
  Vestibular aqueduct (VA) contains the endolymphatic duct and sac. An enlarged VA can be defined using the Valvassori criterion (midpoint diameter > 1.5 mm) [81] or the Cincinnati criteria (midpoint diameter > 1.0 mm or opercular diameter > 2.0 mm) (Fig. 14) [82]. An enlarged vestibular aqueduct (EVA) is associated with a higher risk of intraoperative cerebrospinal fluid gusher and postoperative meningitis [69]. In such cases, careful electrode selection are required to minimise insertion-related complications [79].

## Limitations

Several limitations need to be addressed. Published literatreure on CT imaging parameters is heterogeneous and comprises mainly of retrospective studies with a lack of high-level prospective data and formal consensus guidelines for the suggested thresholds. In addition, there is variability in the CT acquisition protocols, reconstruction techniques, and workstation software across institutions with limited evidence on reproducibility across observers and institutions. Although this article has proposed a standardisation of facial recess width measurements measured on parasagittal reformats, further validation of its consistency is required.

## Conclusion

Pre-operative CT of the temporal bone is important in cochlear implantation for assessing candidacy, surgical risks and guiding electrode insertion. However, variability in acquisition protocols, anatomical definitions, and measurement techniques limits reproducibility of the imaging parameters. This review highlights key surgically relevant CT parameters along the electrode insertion pathway, while proposing a standardised measurement of parasagittal facial recess width, integration of malformation-specific CT assessment, and a structured neuroradiological reporting template as a practical tool for systematic evaluation. 

### Take home messages

- Preoperative temporal bone CT is essential for cochlear implant planning, as it identifies anatomical variations that directly influence surgical approach, feasibility, and risk of complications.
- Key CT parameters include scalp and calvarial thickness, mastoid pneumatisation, tegmen tympani height, vascular anomalies, facial recess width <3 mm which increases risk of facial nerve injury, round window anatomy, and cochlear duct length.
- This review article highlights and addresses areas of ambiguity in the current literature, including inconsistent parameter definitions and a lack of consensus in measurement and scanning techniques. Addressing these gaps with a structured approach is essential for communication with otologists and to ensure accurate surgical planning.

## Data Availability

No datasets were generated or analysed during the current study.

## Appendix

### Example of structured preoperative temporal bone CT report for cochlear implantation

#### Technique

Unenhanced high-resolution CT of temporal bone was performed.

### Findings

#### Right side

The scalp thickness and temporal cortical bone thickness over the anticipated receiver–stimulator site measures __ mm and __ mm respectively.

The mastoid is [well / moderately / poorly pneumatised]. The tegmen tympani height measures __ mm and is [within normal limits / low-lying]. The middle ear and mastoid air cells are [clear / opacified (describe pathology e.g. cholesteatoma if present)].

[There is no vascular anomaly identified.] OR [High-riding jugular bulb (± dehiscence) / Aberrant internal carotid artery / Enlarged mastoid emissary vein measuring __ mm (>3mm) is/are present].

The facial recess width measures __ mm, which is [within normal limits / narrow (<3mm)]. The ossicular chain is [intact / abnormal (describe)].

The round window niche is [normal in size / hypoplastic (<1.5 mm)], [with/without] presence of bony overhang.

The cochlea shows [normal / abnormal morphology (describe types of inner ear malformation; contraindications: rudimentary otocyst, cochlear aplasia)]. The basal turn diameter measures __ mm.

[Additional findings if any]

#### Left side

Same as above

## Impressions

1. [No inner ear malformation] OR [Presence of inner ear malformation(s) including (briefly summarize)].
2. [Unfavourable anatomy for cochlear implants if any]

*Remarks: Bracketed sections are to be selected and modified as appropriate. Sections with “OR” indicate alternative statements depending on imaging findings.*
